# ETS1 Ameliorates Hyperoxia-Induced Bronchopulmonary Dysplasia in Mice by Activating Nrf2/HO-1 Mediated Ferroptosis

**DOI:** 10.1007/s00408-023-00639-1

**Published:** 2023-07-25

**Authors:** Min Yang, Yanping Chen, Xueshan Huang, Fang Shen, Yanni Meng

**Affiliations:** 1 Respiratory Department, Hunan Children’s Hospital, Changsha, 410007 China; 2 University of South China, Hengyang, 421001 China; 3 Research Institute of Children, Hunan Children’s Hospital, Changsha, 410007 China

**Keywords:** Bronchopulmonary dysplasia, ETS1, Nrf2/HO-1, Oxidative stress, Ferroptosis

## Abstract

**Purpose:**

Bronchopulmonary dysplasia (BPD) is associated with hyperoxia-induced oxidative stress-associated ferroptosis. This study examined the effect of E26 oncogene homolog 1 (ETS1) on oxidative stress-associated ferroptosis in BPD.

**Methods:**

Hyperoxia-induced A549 cells and neonatal mice were used to establish BPD models. The effects of ETS1 on hyperoxia-induced ferroptosis-like changes in A549 cells were investigated by overexpression of ETS1 plasmid transfection and erastin treatment. Glucose consumption, lactate production, and NADPH levels were assessed by the glucose, lactate, and NADP^+^/NADPH assay kits, respectively. The potential regulatory relationship between ETS1 and Nrf2/HO-1 was examined by treating hyperoxia-induced A549 cells with the Nrf2 inhibitor ML385. ETS1 effect on the Nrf2 promoter was explored by dual-luciferase reporter and chromatin immunoprecipitation assay. The effect of ETS1 on the symptoms of BPD mice was examined by injecting an adenovirus overexpressing ETS1.

**Results:**

ETS1 overexpression increased hyperoxia-induced cell viability, glucose consumption, lactate production, and NADPH levels and reduced inflammation and apoptosis in A549 cells. In animal experiments, ETS1 overexpression prevented weight loss, airway enlargement, and reductions in radial alveolar counts in BPD mice, while reducing the mean linear intercept, mean alveolar diameter and inflammation. ETS1 overexpression suppressed PTGS2 and CHAC1 expression, reduced ROS, MDA and ferrous iron (Fe^2+^) production and increased GSH levels in hyperoxia-induced A549 cells and BPD mice. In addition, ETS1 can bind to the Nrf2 promoter region and thus promote Nrf2 transcription. ETS1 overexpression increased the mRNA and protein levels of Nrf2, HO-1, xCT, and GPX4 in hyperoxia-induced A549 cells and BPD mice. In hyperoxia-induced A549 cells, erastin and ML385 treatment abolished the effect of ETS1 overexpression.

**Conclusion:**

ETS1 is important in oxidative stress-related ferroptosis in a hyperoxia-induced BPD model, and the effect is partially mediated by the Nrf2/HO-1 axis.

## Introduction

Bronchopulmonary dysplasia (BPD) is the most common and problematic chronic respiratory disease in premature infants. BPD was first identified and named by Northway in 1967 and is defined as disorders of alveolarization, parenchymal fibrosis, and angiogenesis [1]. Although BPD is a multifactorial disease that includes lung immaturity, infection, nutrition, oxygen toxicity, volumetric damage during mechanical ventilation, pneumatic injuries, and inflammatory factor reactions, its main risk factor is due to the effects of exposure to hyperoxia on preterm infants [2, 3]. Hyperoxia causes non-specific changes such as pulmonary edema, inflammation, fibrin deposition, reduced activity of lung surface-active substances and the formation of highly reactive oxygen radicals in the body, triggering oxidative stress [4]. Nuclear factor erythroid-2-related factor 2 (Nrf2) regulates the expression of a large network of genes encoding inducible cytoprotective proteins that enable organisms to adapt and survive under various conditions, including oxidative stress [5]. Nrf2 is associated with its major negative regulatory factor Kelch-like ECH-associated protein 1 (Keap1) together form a molecular effector system that maintains cellular redox homeostasis and thus restores homeostasis in vivo [6]. The antioxidant gene Heme oxygenase-1 (HO-1) is one of the downstream target genes of Nrf2 and is involved in antioxidant stress processes in the organism [7, 8]. In preterm infants, a certain degree of hyperoxia damage can be counteracted by activation of the Nrf2/HO-1 axis [9]. Under normoxic conditions, Nrf2-/- transgenic mice exhibited the same alveolar growth as Nrf2 + / + mice, while under hyperoxic conditions, Nrf2-/- exhibited more severe alveolar growth arrest [9]. Qian et al. showed that administering a single injection of Aurothioglucose (ATG, an FDA-approved thioredoxin reductase inhibitor) to BPD mice to activate Nrf2 activity could inhibit the progression of BPD to some extent [10]. It is clear that Nrf2/HO-1 plays a vital role in counteracting the development of BPD due to mechanical ventilation and oxidative stress. The use of drugs to activate Nrf2/HO-1 can halt the progression of BPD to some extent.

E26 oncogene homolog 1 (ETS1) is an important transcription factor in organisms that regulates biological processes such as cell proliferation, survival, and invasion in vivo by controlling the expression of multiple genes [11]. Earlier research by our group showed that ETS1 protected alveolar epithelial cells from hyperoxia by modulating TGM2 [12]. More research is needed to better understand how ETS1 affects BPD. According to previous reports, ETS1 increases HO-1 expression by activating the HO-1 promoter [13]. We hypothesize that ETS1 can affect BPD progression through Nrf2/HO-1.

Ferroptosis, a form of programmed necrotizing cell death involving iron-dependent lipid peroxidation, plays an important role in BPD's molecular and biological mechanisms and its further development [14, 15]. There is growing evidence for the involvement of Nrf2/HO-1 in ferroptosis [16]. For example, ginsenoside Rg3 was reported to improve severe pancreatitis by triggering the Nrf2/HO-1-mediated ferroptosis pathway [17]. Similarly, inhibiting Nrf2/HO-1 signaling with carboxymethylated pachyman has been shown to induce ferroptosis and treat ovarian cancer [18]. Nrf2 inhibited ferroptosis and prevented acute lung damage caused by intestinal ischemia‒reperfusion by influencing Xc-cystine glutamate transporter (SLC7A11, xCT) and HO-1 [19]. In addition to its regulatory effect on the HO-1 gene, ETS1 has been found to play a role in the synthesis of glutathione (GSH), a key factor in ferroptosis [20]. ETS1 activates the xCT gene promoter and enhances xCT expression, which promotes GSH synthesis by enhancing cellular cystine uptake and enhances GSH/GPX4 antioxidant activity, ultimately enhancing the ability of cells to combat oxidative stress and lipid peroxidation [20, 21]. However, whether ETS1 mediates the regulation of ferroptosis in BPD through Nrf2/HO-1 signaling has not been investigated.

Therefore, the goal of this study was to analyze the expression pattern and function of ETS1 in BPD and determine whether ETS1 ameliorates hyperoxia-induced BPD in mice by activating Nrf2/HO-1-mediated ferroptosis, thus providing a potential target and research foundation for the prevention and cure of BPD.

## Materials and Methods

### Cell Culture

Human alveolar basal epithelial cells (A549, AW-CCH011, Abiowell, China) were cultured in DMEM containing 10% FBS, 100 U/ml penicillin, and 100 μg/ml streptomycin, at 37 °C in 95% air and 5% CO_2_. Cultured A549 cells were subjected to hyperoxia induction and ETS1 overexpression assays [22]. To conduct the hyperoxia experiments, we used subconfluent cells (at 70% with approximately 350–400 cells/mm^2^) and placed them in sealed glass chambers filled with a gas mixture of 95% O_2_ and 5% CO_2_. The cells were then incubated at 37 °C for 48 h. To serve as a control, cells were maintained under normal air conditions, of 21% O_2_ and 5% CO_2_ at 37 °C. The gas and medium were regularly changed every 2 days. To conduct the ETS1 overexpression experiments, we used null/ETS1-overexpression plasmids for transfection. The cells were transfected using Lipofectamine 2000 (Invitrogen, USA) in accordance with the manufacturer’s instructions.

To examine the effect of ETS1 on A549 cells under hyperoxic conditions, the cells were grouped into the Control group, Hyperoxia group, Hyperoxia + oe-NC (ETS1-overexpression negative control) group, and Hyperoxia + oe-ETS1 (ETS1-overexpression) group. The Control group was maintained in normal air. The Hyperoxia group was exposed to hyperoxia, while the Hyperoxia + oe-NC group was transfected with the oe-NC plasmid and then subjected to hyperoxia. The Hyperoxia + oe-ETS1 group was transfected with the oe-ETS1 plasmid and then exposed to hyperoxia.

To investigate the impact of ETS1 on ferroptosis induced by hyperoxia in A549 cells, the cells were grouped into the Control group, Hyperoxia group, Erastin group, Hyperoxia + oe-NC group, Hyperoxia + oe-ETS1 group, and Hyperoxia + oe-ETS1 + Erastin group. Control group cells were kept in normal air conditions. Hyperoxia group cells were exposed to hyperoxia. Cells in the Erastin group were stimulated with 2 μM erastin for 12 h [23] followed by hyperoxia exposure. Hyperoxia + oe-NC group cells were transfected with a null plasmid and then exposed to hyperoxia. Cells in the Hyperoxia + oe-ETS1 group were transfected with an ETS1-overexpression plasmid and then exposed to hyperoxia. Hyperoxia + oe-ETS1 + Erastin group cells were transfected with the ETS1-overexpression plasmid, stimulated with 2 μM erastin for 12 h and finally exposed to hyperoxia.

To test whether ETS1 mediates ferroptosis in A549 cells via the Nrf2/HO-1 axis, cells were grouped into the oe-NC, oe-ETS1, and oe-ETS1 + ML385 groups. oe-NC group cells were transfected with the null plasmid and then exposed to hyperoxia. oe-ETS1 group cells were transfected with ETS1-overexpression plasmid, and then exposed to hyperoxia. oe-ETS1 + ML385 group cells were transfected with the ETS1-overexpression plasmid, stimulated with 5 μM ML385 for 1 h [24] and then exposed to hyperoxia.

### Treatment of Animals

C57BL/6J female mice were ordered from Hunan SJA Laboratory Animal Co., Ltd. The mice delivered naturally on Day 22 of pregnancy. Newborn mice were divided into the Con, Hyperoxia, Hyperoxia + oe-NC, and Hyperoxia + oe-ETS1 groups, with 5 mice in each group. Mice in the Hyperoxia, Hyperoxia + oe-NC and Hyperoxia + oe-ETS1 groups were placed in a sealed plexiglass chamber (22–27 °C, humidity between 50 and 70%) and exposed to hyperoxic conditions (85% oxygen) from Days 1 to 14 after birth [25, 26]. Mice in Con group were left in normal room air (21% oxygen). In addition, mice in the Hyperoxia + oe-NC and Hyperoxia + oe-ETS1 groups were intraperitoneally injected with 30 μL of oe-NC/oe-ETS1 lentivirus (1 × 10^8^ TU/ml) every 3 days on postnatal Days 4–14 [24, 27]. The mice in the Con and Hyperoxia groups were injected with 0.9% NaCl. Neonatal mice were euthanized on postnatal Day 14, and blood, bronchoalveolar lavage fluid (BALF), and lung tissues were obtained for further analysis.

### Histological Tests (Hematoxylin–Eosin Staining)

The lung tissues were coated with paraffin, baked, dewaxed, and hydrated with xylene. Hematoxylin and eosin (Abiowell, China) were used to stain the sections. After gradient ethanol dehydration and xylene removal, neutral rubber was used for sealing. Under a light microscope (BA210T, Motic), morphological alterations in lung tissue were examined and assessed.

The radial alveolar count (RAC) was determined by counting the number of alveoli intersecting a vertical line from the terminal bronchial margin to the closest pleura or mediastinum. The mean alveolar diameter (MAD) was calculated on the basis of the average diameter of the alveoli. Five randomly selected lines were used in each region to measure the mean linear intercept (MLI), which was the number of alveoli crossing each line.

### Quantitative Reverse Transcription PCR (RT‒qPCR)

Total RNA was extracted from lung tissues and cells with the Trizol (15596026, Thermo) method. The obtained RNA was then used as a template for cDNA synthesis using the RNA Reverse Transcription Kit (CW2569, CWbio). The resulting cDNA was amplified with UltraSYBR Mixture (CW2601, CWbio), and fluorescent signals were detected in real-time. The amplification conditions included 10 min at 95 °C for initialization, followed by denaturation at 95 °C for 15 s and 60 °C for 30 s with 40 cycles. Gene expression was calculated using the 2^−ΔΔCt^ method, and β-actin served as an internal reference. Table 1 lists the primer sequences.

**Table 1:** Primer sequences

Gene	Sequences (5'-3')
β-actin-Human	F: ACCCTGAAGTACCCCATCGAG
	R: AGCACAGCCTGGATAGCAAC
ETS1-Human	F: CCACAGACTTTGAGGGAAGC
	R: CTGCTCTCAGCACCTCACTT
PTGS2-Human	F: CTGCGCCTTTTCAAGGATGG
	R: CCCCACAGCAAACCGTAGAT
CHAC1-Human	F: AGTGCAAGGGGAGCAGAACC
	R: TGCCAGACGCAGCAAGTATT
Nrf2-Human	F: CAACTCAGCACCTTATATCTCG
	R: ACAAGGAAAACATTGCCATC
HO-1-Human	F: CACACCCAGGCAGAGAATGCT
	R: GGCTCTCCTTGTTGCGCTCA
xCT-Human	F: CTCCAGGTTATTCTATGTTGCGTCT
	R: CAAAGGGTGCAAAACAATAACAGC
GPX4-Human	F: CGCCTTTGCCGCCTACTGAAGC
	R: AACCATGTGCCCGTCGATGTCC
β-actin-mouse	F: ACATCCGTAAAGACCTCTATGCC
	R: TACTCCTGCTTGCTGATCCAC
ETS1-mouse	F: AGTTTCAGCCATCACAACACA
	R: GAAATCCTACCTGACGAGCAC
PTGS2-mouse	F: AATACTGGAAGCCGAGCACCT
	R: ACACCCCTTCACATTATTGCAGA
CHAC1-mouse	F: GCCCTGTGGATTTTCGGGTA
	R: CGGTCTTCAAGGAGGGTCAC
Nrf2-mouse	F: GCTCCTATGCGTGAATCCCAA
	R: TTTGCCCTAAGCTCATCTCGT
HO-1-mouse	F: TCCATGTTGACTGACCACGACT
	R: CCCACCCCTCAAAAGATAGCC
xCT-mouse	F: CATACTCCAGAACACGGGCAG
	R: AACAAAAGCCAGCAAAGGACCA
GPX4-mouse	F: CATCGACGGGCACATGGTCT
	R: CCACACTCAGCATATCGGGCAT

### Western Blot

RIPA lysis buffer (AWB0136, Abiowell) was used to extract total proteins from lung tissues and A549 cells. SDS‒PAGE was used to separate the proteins, which were then transferred to nitrocellulose filter membranes. Nonspecific antigens were blocked using 5% skimmed milk. Next, the membranes were treated with primary antibodies overnight at 4 °C. The secondary antibody was then added and incubated with the membranes at 25 °C for 1.5 h. Information on the antibodies is listed in Table 2. ECL chemiluminescent solution (AWB0005, Abiowell) was added and incubated with the membranes for 1 min, and pictures of the target bands were obtained in a chemiluminescent imaging system (ChemiScope 6100, Clinx). The internal reference was β-actin. The grayscale values of the target bands were examined using Image J.

**Table 2:** Antibody information

Name	Dilution rate	Cat number	Source	Company	Country
ETS1	1: 500	12118-1-AP	Rabbit	Proteintech	USA
PTGS2	1: 3000	ab179800	Rabbit	Abcam	UK
CHAC1	1: 1000	15207-1-AP	Rabbit	Proteintech	USA
Nrf2	1: 5000	16396-1-AP	Rabbit	Proteintech	USA
HO-1	1: 3000	10701-1-AP	Rabbit	Proteintech	USA
xCT	1: 1000	26864-1-AP	Rabbit	Proteintech	USA
GPX4	1: 1000	67763-1-Ig	Mouse	Proteintech	USA
β-actin	1: 5000	66009-1-Ig	Mouse	Proteintech	USA
HRP goat anti-Rabbit IgG	1: 6000	SA00001-2	–	Proteintech	USA
HRP goat anti-mouse IgG	1: 5000	SA00001-1	–	Proteintech	USA

### Cell Counting Kit-8 (CCK-8) Assay

A549 cells were seeded in 96-well plates at a density of 1 × 10^4^ cells per well and incubated for 12 h at 37 °C in a 5% CO_2_ incubator. Ten microliters of CCK-8 reagent was added to each well in 100 μL of fresh medium. The plates were then incubated for 4 h. The optical density (OD) value of the samples was measured at 450 nm using a microplate reader (MB-530, HEALES).

### Flow Cytometry

Apoptosis was detected in A549 cells using an Apoptosis Detection Kit (APC, KGA1030, KeyGEN BioTECH). Approximately 3.2 × 10^5^ cells were collected and resuspended in 500 μL of binding buffer. Then, 5 μL of Annexin V-FITC and 5 μL of propidium iodide were added to each sample and incubated for 10 min. The percentage of apoptotic cells was analyzed by a flow cytometer (A00-1-1102, Beckman).

### Glucose Consumption, Lactate Production, and NADPA Level Assays

The glucose concentration in the collected medium was assessed using a glucose assay kit (A154-1-1, Nanjing Jiancheng Bioengineering Institute) according to the manufacturer's instructions. Glucose consumption was calculated by measuring the concentration of glucose in the original fresh medium minus the concentration of glucose in the medium collected at the specified time. Similarly, lactate production in the medium was determined by the Lactate Assay Kit (A019-2–1, Nanjing Jiancheng Bioengineering Institute). The lactate production was calculated by subtracting the lactate concentration in the original fresh medium from the lactate concentration in the medium collected at the specified time. Intracellular NADPH levels were measured using the NADP^+^/NADPH assay kit (S0179, Beyotime Biotechnology) according to the manufacturer's instructions.

### ROS Detection

The cells were treated with 0.25% trypsin, and the resulting cell suspension was collected. After centrifugation at 2000 rpm for 5 min, the supernatant was discarded and the cells were washed twice with PBS. The cells were then incubated with 5 μM C11-BODIPY (MX5211-1MG, Shanghai Maokang Biotechnology Co., Ltd.) at 37 °C for 1 h. ROS levels in the cells were detected using a flow cytometer (A00-1-1102, Beckman).

### Detection of GSH, MDA, and Ferrous Iron (Fe^2+^) Levels

GSH (A006-2, Nanjing Jiancheng Bioengineering Institute), MDA (A003-1, Nanjing Jiancheng Bioengineering Institute), and iron (ab83366, Abcam) assay kits were used to measure the levels of GSH, MDA, and Fe^2+^ in the cells, respectively. The concentrations of GSH, MDA, and Fe^2+^ were calculated based on the standard curves provided by Bio-Tek (MB-530, Heales, China).

### Enzyme-Linked Immunosorbent Assay (ELISA)

TNF-α, IL-6, and IL-1β levels in BALF were determined following the guidelines provided by the TNF-α (KE10002, Proteintech), IL-6 (KE10007, Proteintech) and IL-1β (CSB-E08054m, CUSABIO) ELISA kits.

### Dual-Luciferase Reporter Assay

Human wild-type Nrf2 promoter region gene dual luciferase reporter plasmid (Nrf2-WT, 5′- AACGCCTTTCCGGGGCTCC -3′) and human mutant type Nrf2 promoter region gene dual luciferase reporter plasmid (Nrf2-MUT, 5′- AACGCCAAAGGCGGGCTCC -3′), ETS1 gene overexpression plasmid and its control plasmid were purchased from HonorGene (Abiowell, China). Lipofectamine 2000 reagent (Invitrogen) was used to cotransfect the above plasmids into 293T cells (Abiowell, China). After 48 h of transfection, the luciferase activity was performed by a dual-luciferase assay kit (E1910, Promega, USA).

### Chromatin Immunoprecipitation (CHIP) Assay

CHIP assay was performed using a CHIP Assay Kit (ab500, Abcam) [28]. Chromatin was cross-linked with 1.1% formaldehyde and after neutralization with glycine. Cells were centrifuged at 4 °C, 500 g for 5 min, and cell sediments were collected. Cells were resuspended with 1.1% formaldehyde, Buffer A, and PBS, then neutralization with glycine. Cells were resuspended sequentially with Buffer B and Buffer C. After centrifugation at 4 °C, 500 g for 5 min and removal of the supernatant, cells were resuspended with Buffer D and protease inhibitor mixture. Then the cells were sonicated and broken (sonication 4 s, gap 6 s, total time 60 s, power 20%). After centrifugation at 4 °C, 14,000 g for 5 min, the supernatant was obtained. The supernatant was added to 1 × CHIP buffer. Chromatin was immunoprecipitated with ETS1 (ab307672, Abcam), Positive control (ab1791, Abcam), and Negative control (IgG, Proteintech, 30000-0-AP) antibody at 4 °C. The immunoprecipitation products were collected after incubation with G protein agarose beads. 100 µL of DNA purifying slurry was resuspended on agarose agglutinates, incubated at 98 °C for 10 min. 10,000 g centrifugation was performed for 10 s to remove the agarose agglutinates. Then the supernatant was added 1 µL of proteinase K with digestion at 55 °C for 30 min and incubated at 98 °C for 10 min. Centrifugation (14,000 g, 1 min) to obtain the supernatant. After DNA purification, evaluate the Nrf2 promoter binding site by RT-qPCR. Primers were shown in Table 3.

**Table 3:** Primer sequences

Gene	Sequences (5'-3')
Nrf2-CHIP1	F: CTAAACCAAATGCCAAAC
	R: TTCACTTTTCCAATGCTG
Nrf2-CHIP2	F: CAGCATTGGAAAAGTGAA
	R: GAGCCTAGTAGGTAGGAGAT
Nrf2-CHIP3	F: CGGGCGGTAAAGTGAGAT
	R: AAAGAGTTGTTTGCGAAGGTC

### Statistical Analysis

All data are expressed as the mean ± SD. Unpaired t tests were used to analyze the significance between two groups, while one-way analysis of variance (ANOVA) followed by a post hoc test was used for multiple comparisons involving three or more groups. GraphPad Prism 8.0 software was used for statistical calculations. A p value less than 0.05 indicated a significant difference between groups.

## Results

### ETS1 Inhibits Hyperoxia-Induced Ferroptosis in A549 Cells

**Fig. 1:**
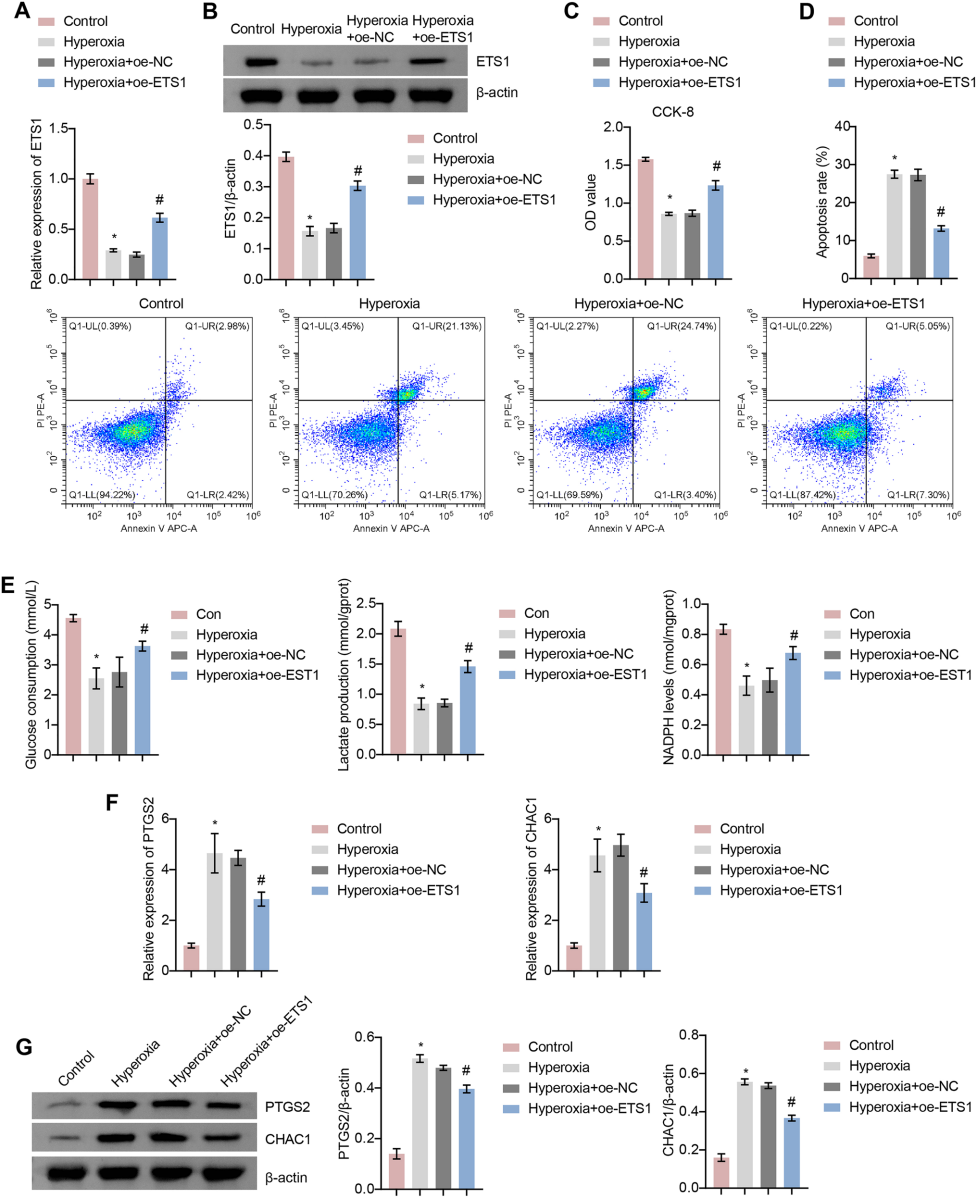
ETS1 inhibits hyperoxia-induced ferroptosis in A549 cells. **A** RT‒qPCR verification of ETS1 levels. **B** Western blot analysis of ETS1 expression. **C** CCK-8 assay results. **D** Flow cytometry was used to identify cell apoptosis. **E** Glucose consumption, lactate production, and NADPH levels. **F** RT‒qPCR verification of PTGS2 and CHAC1 expression. **G** Western blot analysis of PTGS2 and CHAC1 levels. ^*^ vs. Control. ^#^ vs. Hyperoxia + oe-NC. *P* < 0.05

To investigate the role of ETS1 in BPD, we transfected ETS1-overexpression plasmids into hyperoxia-induced A549 cells. ETS1 mRNA and protein levels were consistently reduced in hyperoxia-induced A549 cells. After transfection with the ETS1-overexpression plasmid, ETS1 levels were increased in hyperoxia-induced A549 cells (Fig. 1A and B). The CCK-8 assay showed an increase in OD values in the Hyperoxia group compared to the Control group, suggesting that hyperoxia exposure decreased cell activity (Fig. 1C). Flow cytometry unraveled that by contrast to the Control group, the Hyperoxia group had a distinct decline in cell apoptosis (Fig. 1D). In contrast, ETS1 overexpression reversed the effects of hyperoxia on A549 cells (Fig. 1C and D). Glucose consumption, lactate production, and NADPH levels were decreased in A549 cells after hyperoxia treatment. This change was reversed by ETS1 overexpression (Fig. 1E). The levels of the ferroptosis-related proteins PTGS2 and CHAC1 were further examined. The results demonstrated that hyperoxia increased PTGS2 and CHAC1 expression in A549 cells, and the ETS1 overexpression treatment reversed this change (Fig. 1F and G). These results indicate that ETS1 inhibits hyperoxia-induced ferroptosis in alveolar epithelial cells and increases cellular activity and glycolytic capacity.

### ETS1 Reduces Hyperoxia-Induced Oxidative Stress and Inflammation in A549 Cells by Mediating Fe^2+^ Enrichment

**Fig. 2:**
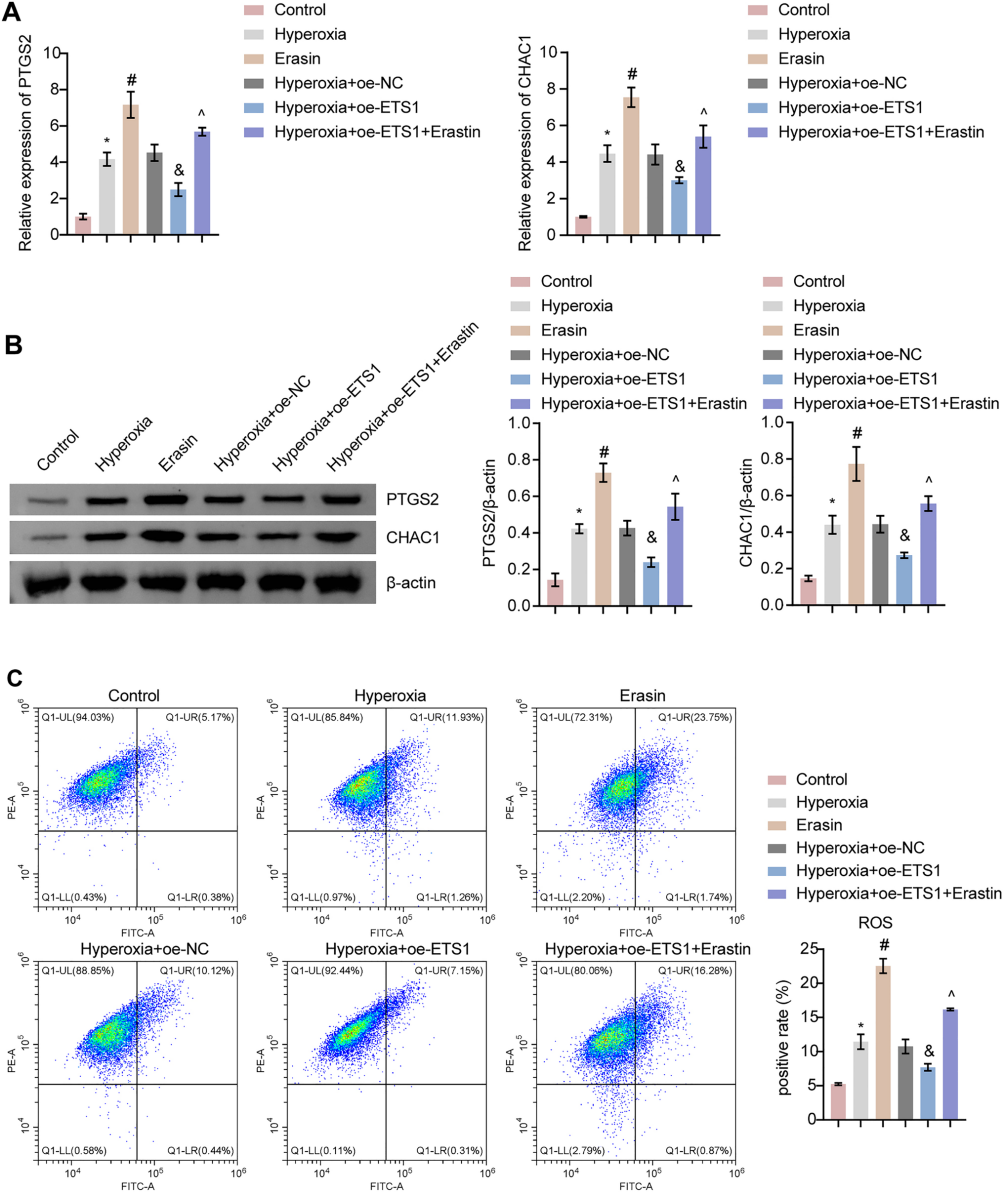

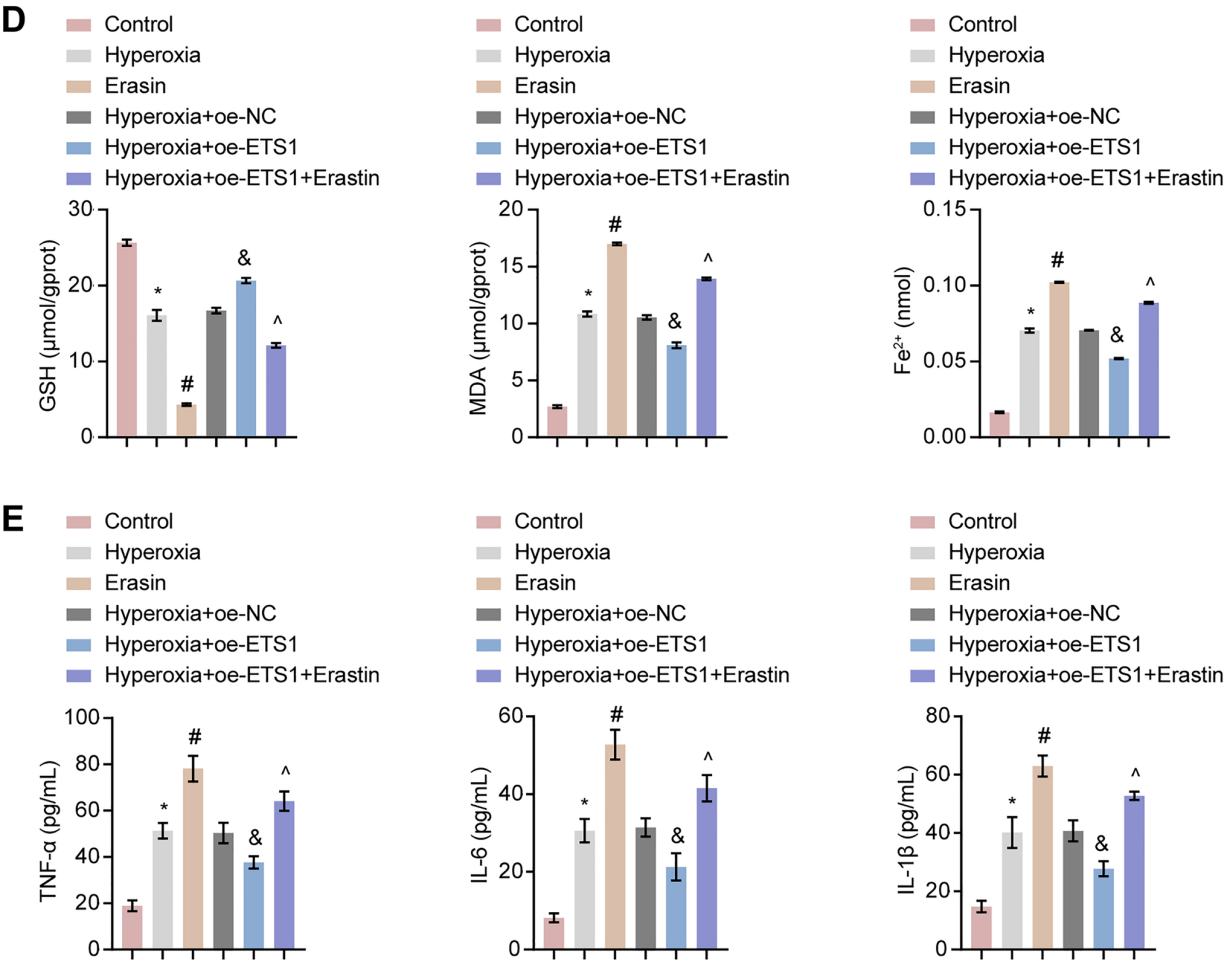
ETS1 reduces hyperoxia-induced oxidative stress and inflammation in A549 cells by mediating Fe^2+^ enrichment. **A**, **B** RT‒qPCR (**A**) and Western blot analysis (**B**) of PTGS2 and CHAC1 levels. **C** The formation of ROS in A549 cells was measured using the C11-BODIPY fluorescent probe. **D** The concentrations of GSH, MDA and Fe^2+^ were assessed in A549 cells. **E** TNF-α, IL-6, and IL-1β levels in A549 cells were tested. * vs. Control. ^#^ vs. Hyperoxia. ^&^ vs. Hyperoxia + oe-NC. ^^^ vs. Hyperoxia + oe-ETS1. *P* < 0.05

To better understand the role of ETS1 in hyperoxia-induced ferroptosis in A549 cells, we treated ETS1-overexpressing A549 cells with the ferroptosis-inducing agent erastin. The results showed that there was a significant increase in PTGS2 and CHAC1 levels in the Hyperoxia + oe-ETS1 + Erastin group compared to the Hyperoxia + oe-ETS1 group (Fig. 2A and B). We then examined the levels of ROS, GSH, MDA, and Fe^2+^. In A549 cells, hyperoxia increased ROS, MDA, and Fe^2+^ levels and reduced GSH levels (Fig. 2C and D). In contrast, ETS1 overexpression considerably reduced hyperoxia-induced ROS, MDA, and Fe^2+^ levels while simultaneously increasing GSH levels. However, in the presence of erastin, the impact of ETS1 overexpression on hyperoxia-induced oxidative stress in A549 cells was reversed (Fig. 2C and D). Hyperoxia also increased inflammatory factor levels (TNF-α, IL-6, and IL-1β) in A549 cells, which was reversed by ETS1 overexpression (Fig. 2E). Similarly, erastin treatment negated the effect of ETS1 overexpression on hyperoxia-induced inflammation in A549 cells (Fig. 2E).

### ETS1 Activates the Hyperoxia-Induced Nrf2/HO-1 Axis in A549 Cells

**Fig. 3:**
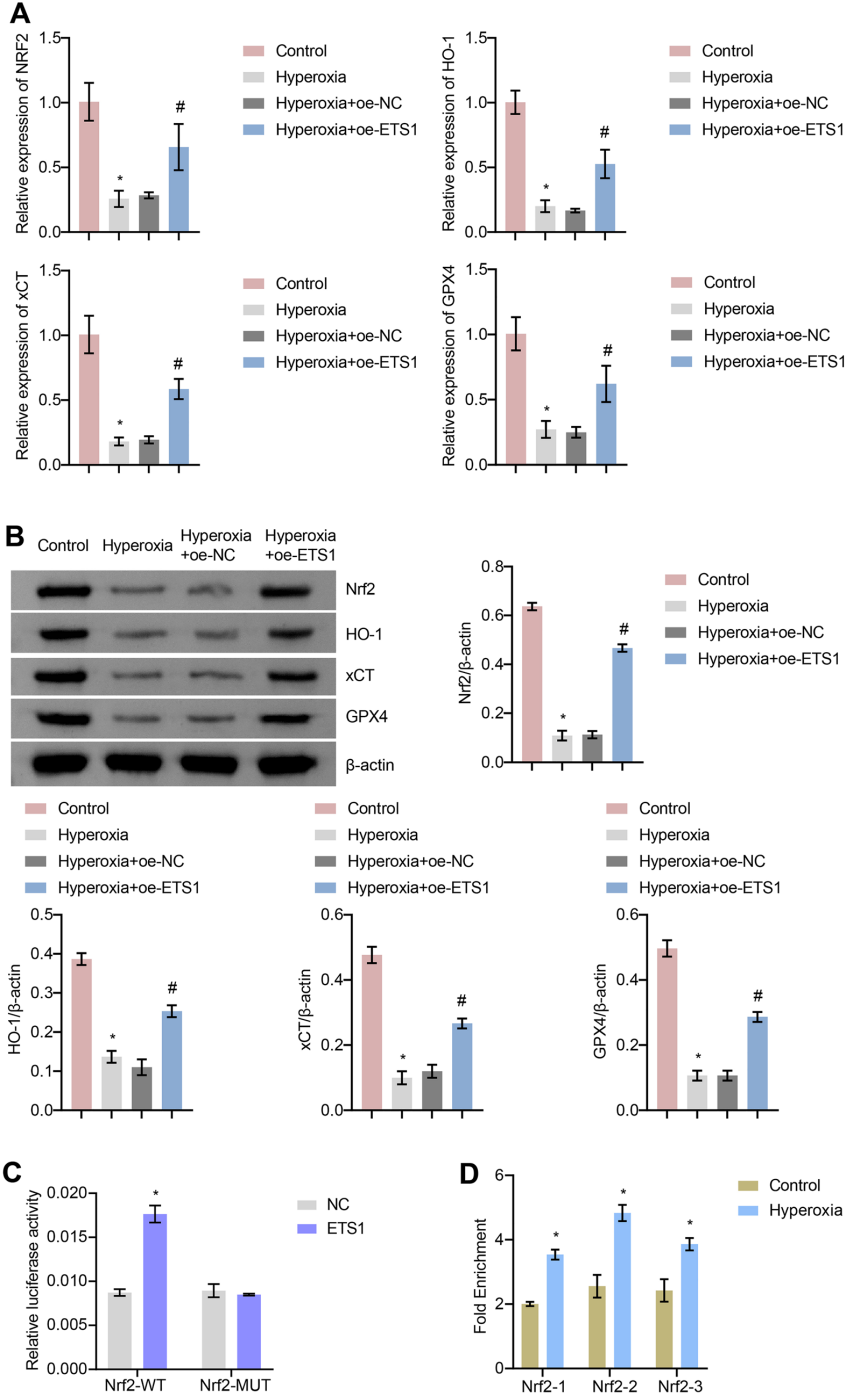
ETS1 activates the hyperoxia-induced Nrf2/HO-1 axis in A549 cells. **A** RT‒qPCR analysis of Nrf2, HO-1, xCT, and GPX4 levels in cells. **B** Western blot analysis of Nrf2, HO-1, xCT, and GPX4 protein levels in cells. **C** Nrf2 promoter dual-luciferase reporter assay. **D** CHIP assay demonstrated that ETS1 bound to the Nrf2 promoter. * vs. Control or NC. ^#^ vs. Hyperoxia + oe-NC. *P* < 0.05

By using RT‒qPCR and Western blot, we investigated how ETS1 affected the Nrf2/HO-1 axis in A549 cells. Nrf2, HO-1, xCT, and GPX4 levels were reduced in the Hyperoxia group compared to the Control group (Fig. 3A and B). ETS1 overexpression increased the mRNA and protein levels of Nrf2, HO-1, xCT, and GPX4 in hyperoxia-induced cells. After JASPAR database analysis, it was found ETS1 protein's binding location in the Nrf2 promoter. The effect of ETS1 on Nrf2 promoter activity was verified by a dual-luciferase reporter assay. The results showed that ETS1 overexpression increased luciferase activity in the Nrf2-WT group, but not in the Nrf2-MUT group (Fig. 3C). Further results from CHIP assay showed that ETS1 bound directly to the Nrf2 promoter region (Fig. 3D). The above results suggest that ETS1 can bind to the Nrf2 promoter region, thus promoting Nrf2 transcription and affecting the Nrf2/HO-1 pathway.

### The Nrf2 Inhibitor ML385 Abolishes the Impact of ETS1 on A549 Cells Subjected to Hyperoxia

**Fig. 4:**
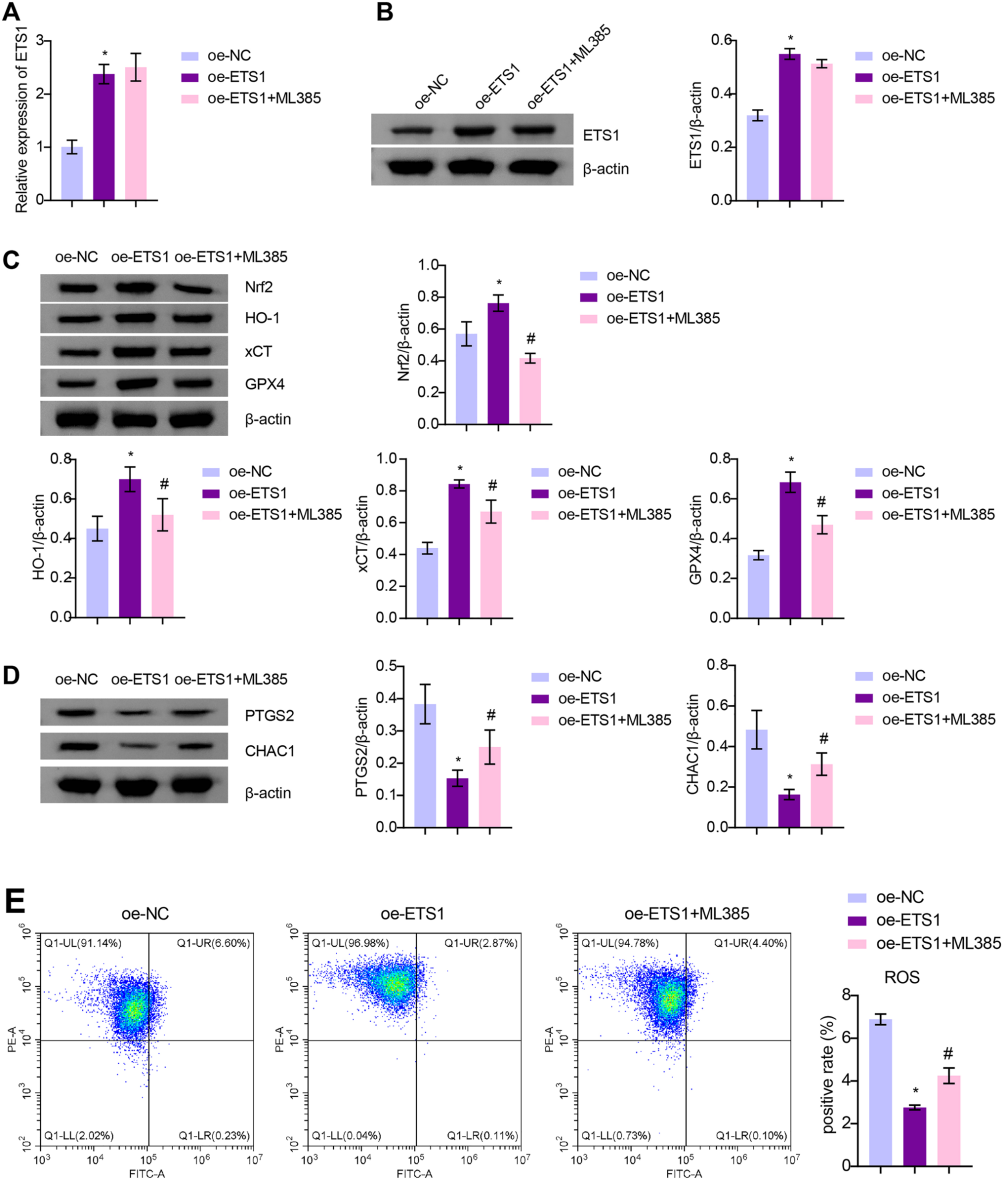

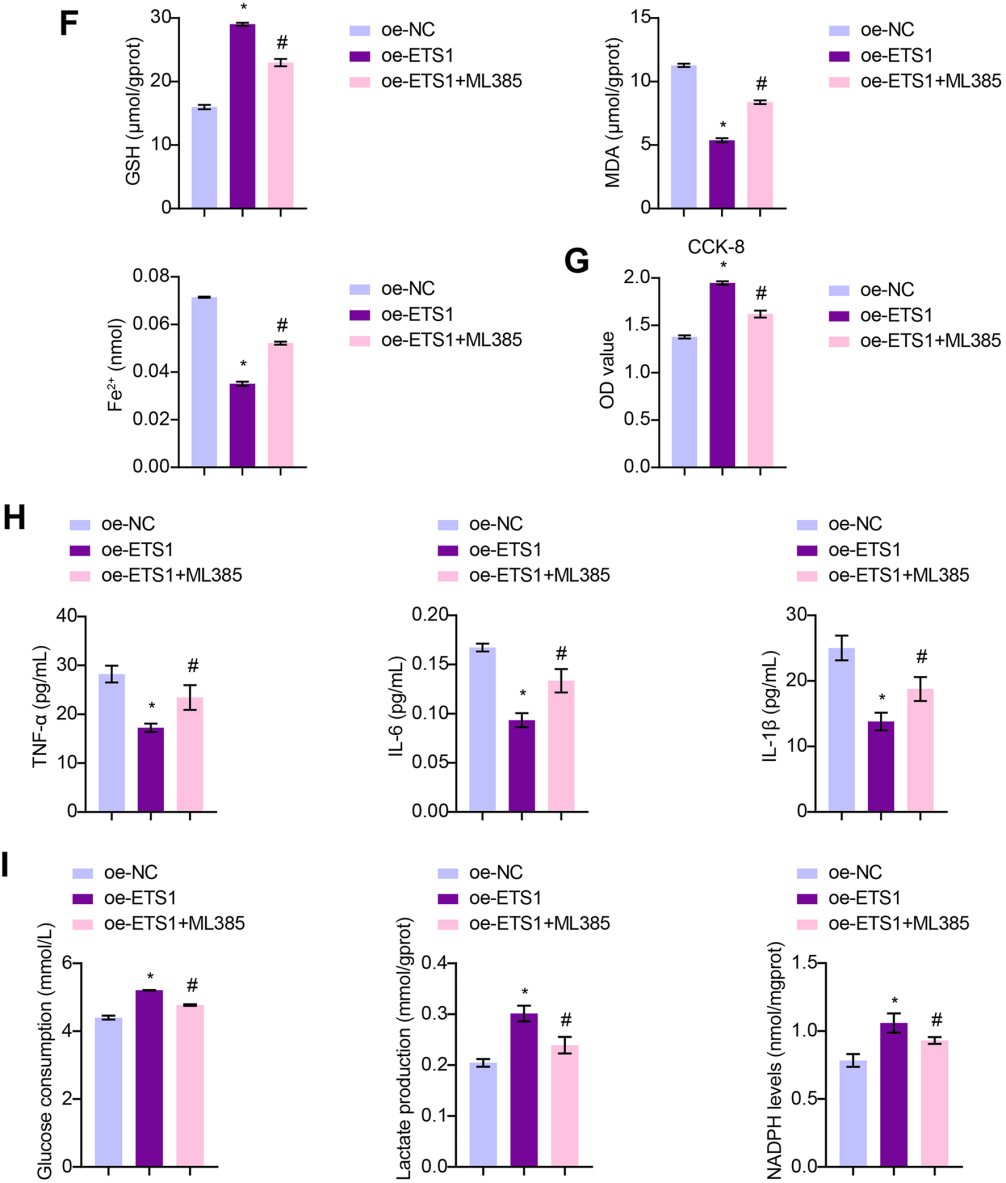
The Nrf2 inhibitor ML385 abolishes the impact of ETS1 on A549 cells subjected to hyperoxia. RT‒qPCR (**A**) and Western blot (**B**) analysis of ETS1 levels. **C** Western blot analysis of Nrf2, HO-1, xCT, and GPX4 levels in A549 cells. **D** Western blot analysis of PTGS2 and CHAC1 expression. ROS production (**E**) and GSH, MDA, and Fe^2+^ levels (**F**) in cells. **G** CCK-8 results. **H** TNF-α, IL-6, and IL-1β levels in A549 cells were tested. **I** Glucose consumption, lactate production, and NADPH levels. * vs. oe-NC. ^#^ vs. oe-ETS1. *P* < 0.05

To investigate the role of Nrf2/HO-1 in mediating the effects of ETS1 on A549 cells, we treated ETS1-overexpressing cells with hyperoxia and the Nrf2 inhibitor, ML385. Interestingly, ML385 treatment did not affect ETS1 expression in the ETS1-overexpressing cells (Figs. 4A and B). However, Nrf2, HO-1, xCT, and GPX4 levels were reduced in ETS1-overexpressing cells that were treated with ML385 (Fig. 4C). Similarly, the ETS1-overexpressing cells had lower levels of PTGS2 and CHAC1 than those in the oe-NC group, however treatment with ML385 restored this change (Fig. 4D). Furthermore, when we analyzed the cellular levels of ROS, MDA, Fe^2+^, and GSH, we noted that ML385 treatment effectively reversed the decrease in ROS, MDA, and Fe^2+^ and decreased GSH levels in ETS1-overexpressing cells (Fig. 4E and F). Likewise, ML385 treatment reversed the increase in cell viability and the decrease in inflammation in ETS1-overexpressing cells (Fig. 4G and H). Glucose consumption, lactate production, and NADPH levels were increased in EST1 overexpressing cells, and this change was reversed by ML385 treatment (Fig. 4I). Overall, these findings indicate that the Nrf2/HO-1 axis is important for mediating the effects of ETS1 on A549 cells.

### ETS1 Overexpression Improves BPD Symptoms

**Fig. 5:**
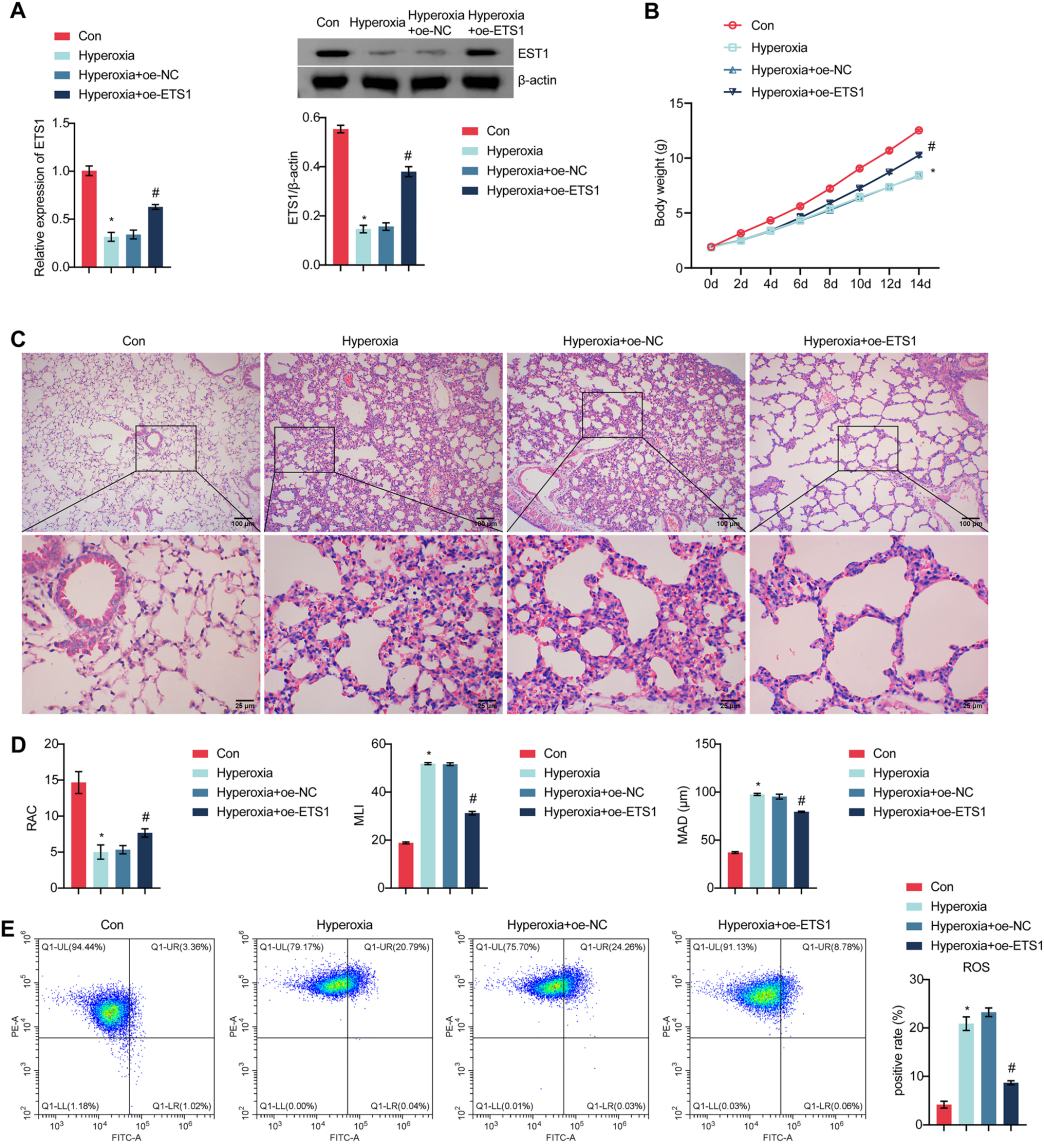

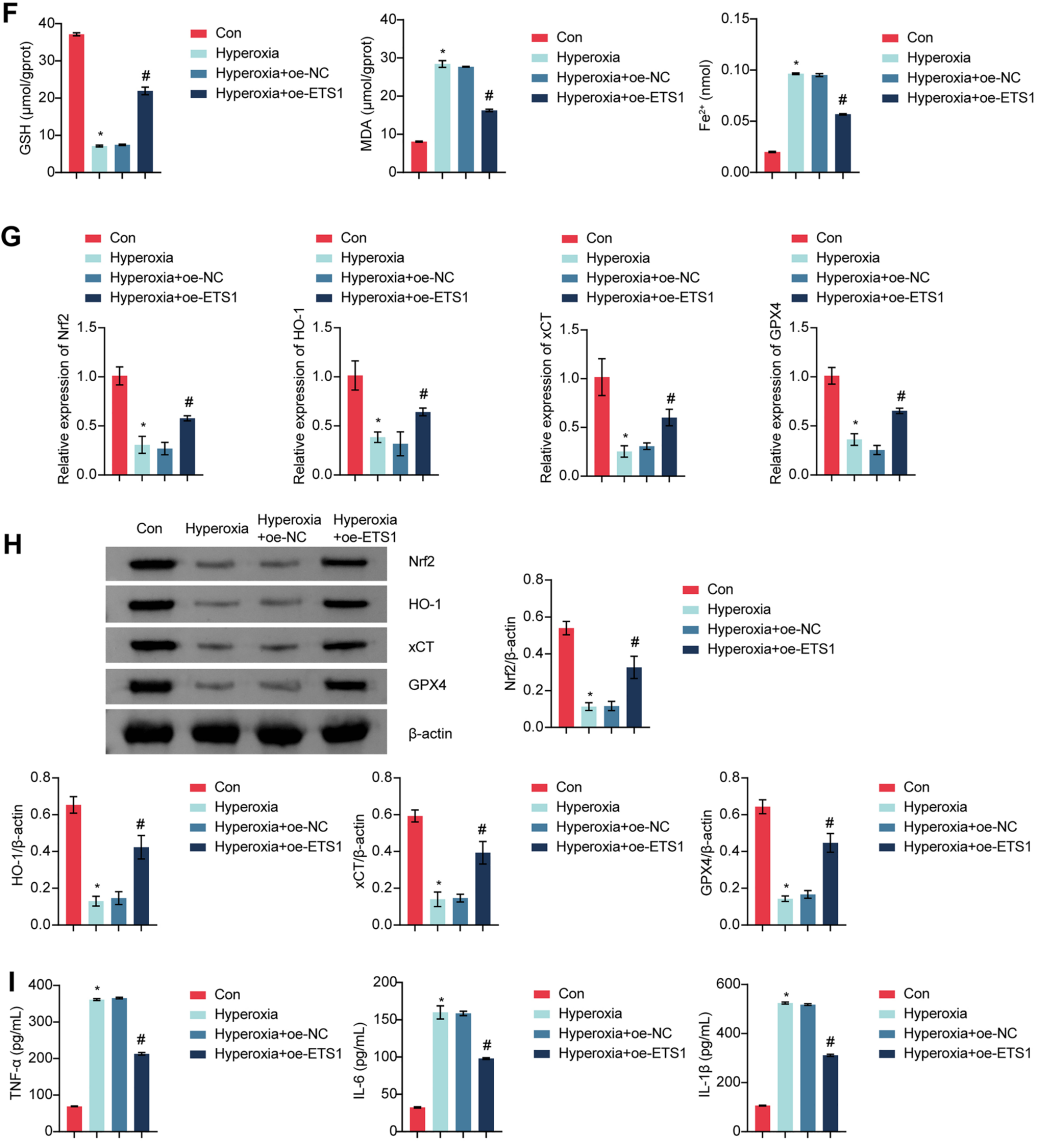
ETS1 overexpression improves BPD symptoms. **A** RT‒qPCR and Western blot analysis of ETS1 levels in lung tissue. **B** Weight monitoring. **C** Observation of lung histomorphology. **D** RAC, MLI, and MAD evaluation. ROS production (**E**) and GSH, MDA, and Fe^2+^ levels (**F**) in lung tissue. **G** RT‒qPCR analysis of Nrf2, HO-1, xCT, and GPX4 expression in lung tissue. **H** Western blot analysis of Nrf2, HO-1, xCT, and GPX4 protein levels in lung tissue. **I** TNF-α, IL-6, and IL-1β levels in BALF. * vs. Con. ^#^ vs. Hyperoxia + oe-NC. *P* < 0.05

To study the effect of ETS1 on BPD symptoms, we created a mouse BPD model. Figure 5A shows that in comparison to that in the Con group, ETS1 expression was considerably lower in hyperoxia-exposed mice. Injection of the ETS1 overexpression adenovirus increased ETS1 levels in hyperoxia-exposed mice. Body weight was lower in hyperoxia-exposed mice than in Con mice, and ETS1 overexpression prevented weight loss in hyperoxia-exposed mice (Fig. 5B). Hematoxylin–eosin staining showed airway enlargement in hyperoxia-exposed mice, which was prevented by ETS1 overexpression (Fig. 5C). Hyperoxia exposure reduced the RAC in mice and increased MLI and MAD compared to those in the Con group. However, ETS1 overexpression reversed this change (Fig. 5D). ROS, MDA and Fe^2+^ levels were increased, and GSH levels were decreased in hyperoxia-exposed mice compared to those in the Con group. ETS1 overexpression reversed the hyperoxia-induced increase in ROS, MDA, and Fe^2+^ levels and increased GSH levels (Fig. 5E and F). Nrf2, HO-1, xCT, and GPX4 expression was downregulated in lung tissue in the Hyperoxia group compared to the Con group (Fig. 5G and H). Nrf2, HO-1, xCT, and GPX4 expression was increased in the Hyperoxia + oe-ETS1 group relative to the Hyperoxia + oe-NC group. ELISA results showed elevated levels of TNF-α, IL-6, and IL-1β in the Hyperoxia group relative to the Con group, and this effect was reversed by oe-ETS1 treatment (Fig. 5I). These results demonstrate that ETS1 overexpression substantially alleviated BPD symptoms in hyperoxia-exposed neonatal mice.

## Discussion

We systematically investigated the association between ETS1 expression and BPD progression. Our research revealed that ETS1 expression was low in hyperoxia-exposed A549 cells and neonatal mice. We also discovered that ETS1 overexpression effectively suppressed ferroptosis in hyperoxia-exposed A549 cells. Moreover, the Nrf2/HO-1 pathway is a key downstream component of ETS1 in the context of BPD progression. Overall, our study identified a promising novel ETS1 signaling pathway that might be a viable target for BPD treatment.

BPD is a multifactorial disease, and the most significant influence is oxidative stress caused by hyperoxia [29, 30]. When the oxidative stress balance in the body is disrupted, it induces different forms of cell death, including ferroptosis [31]. Ferroptosis is a type of programmed cell death that is induced by lipid peroxidation and depends on iron. Previous studies have suggested that ferroptosis contributes to hyperoxic lung injury in neonatal rats [32]. Our current research showed that similar ferroptosis-like changes occurred in the alveolar epithelial cells and lung tissue of mice in the BPD group that were exposed to high oxygen levels. These changes included increases in Fe^2+^, ROS, and MDA levels, as well as elevated expression of the genes PTGS2 and CHAC1, and reduced levels of the antioxidant GSH.

ETS1 has been reported to regulate ferroptosis as a transcription factor in disease states [33]. For example, ETS1 promotes resistance to sorafenib in hepatocellular carcinoma by regulating ferroptosis via the miR-23a-3p/ACSL4 axis [34]. In the present study, cells in the ETS1 overexpression group showed increased viability and reduced apoptosis compared to those treated with hyperoxia. Ferroptosis is induced by increases in lipid ROS production, and intracellular concentrations of reduced iron ions and reduced levels of the antioxidant GSH [35]. ETS1 overexpression reduced hyperoxia-induced ROS, MDA, and Fe^2+^ production and increased GSH levels. ETS1 overexpression inhibited PTGS2 and CHAC1 expression in hyperoxia-induced alveolar epithelial cells, and the effects of ETS1 overexpression were reversed by the ferroptosis inhibitor erastin. In addition, ETS1 overexpression improved the symptoms of BPD mice and prevented airway enlargement, weight loss, increased inflammation, and ferroptosis-like changes in hyperoxia-exposed mice. These observations suggest that the protective effects of ETS1 on experimental models of hyperoxia-induced BPD in vivo and ex vivo may be mediated through its effects on ferroptosis.

Hyperoxia reduces the determined glycolysis, glycolytic reserve or glycolytic capacity of lung epithelial cells [36]. Glucose supplementation prevented extensive death of cultured human lung epithelial-like cells under hyperoxic conditions [37]. Our study found that ETS1 overexpression reversed hyperoxia-induced reductions in glucose depletion, lactate production, and NADPH production in alveolar epithelial cells. ETS1 prevents hyperoxia injury by activating the Warburg effect in cells [38, 39], which is consistent with our results. In addition Nrf2 inhibitor ML385 treatment blocked the protective effect of ETS1 overexpression on glycolysis in hyperoxia-treated lung epithelial cells. These results suggest that ETS1 plays a protective role in preserving cellular glycolytic capacity through the Nrf2/HO-1 pathway, thereby shielding cells from damage caused by hyperoxia.

Previous studies have shown that Nrf2 plays a critical role in mediating lipid peroxidation and ferroptosis [40, 41]. Nrf2/HO-1 axis activation can reduce LPS-induced acute lung injury by inhibiting ferroptosis [16]. xCT and GPX4 are two of the most critical targets that regulate ferroptosis, and the inhibition of these factors triggers ferroptosis, which has been reported to be regulated by Nrf2 [42, 43]. Inhibiting Nrf2 has also been shown to reduce antioxidant capacity and induce ferroptosis by inhibiting GPX4 expression in models of oxygen–glucose deprivation/reperfusion-induced neuronal injury [44]. The activation of Nrf2/HO1 in sustained oxidative stress-induced lung injury in preterm infants is consistent with the induction of a certain amount of antioxidant and anti-inflammatory activity [45]. Moreover, activation of the Nrf2-ARE pathway attenuates abnormal alveolarization in neonatal rats with BPD [46]. Nrf2 was identified as one of the major downstream targets of ETS1 in our study. Our results show that ETS1 can bind to the Nrf2 promoter region and thus promote Nrf2 transcription. The reduction in Nrf2, HO-1, GPX4, and xCT levels induced by hyperoxia could be reversed by ETS1 overexpression. Meanwhile, treatment with the Nrf2 inhibitor ML385 blocked the protective effect of ETS1 overexpression on ferroptosis in hyperoxia-induced lung epithelial cells. Thus, ETS1 could protect lung epithelial cells and neonatal mice from hyperoxia-induced ferroptosis by activating the cellular Nrf2/HO-1 axis.

## Conclusion

This study’s findings demonstrate that ETS1 plays a protective role in BPD by suppressing oxidative stress-induced ferroptosis. This effect is achieved by activating the Nrf2/HO-1 axis. To our knowledge, this is the first study to report the role of ETS1 in BPD with respect to ferroptosis regulation.

## Data Availability

All data were displayed in the article.
